# A Scoping Review on Community-based Diabetes Screening Interventions: Paving the Pathway to Early Care and Prevention of Diabetes

**DOI:** 10.1007/s11892-025-01605-2

**Published:** 2025-10-04

**Authors:** Aimen Zehra, David Gerstle, Fatema M. Ali, Muhanad Ali, Cilia Mejia-Lancheros, Ghazal S. Fazli

**Affiliations:** 1 Department of Geography, Geomatics, and the Environment, University of Toronto Mississauga, Mississauga, ON Canada; 2 University of Toronto Mississauga Library, Mississauga, ON Canada; 3 Network for Healthy Populations, University of Toronto, Toronto, ON Canada; 4 University of Toronto, Toronto, ON Canada; 5 Institute for Better Health, Trillium Health Partners, Mississauga, ON Canada; 6 Institute for Clinical Evaluative Sciences, Toronto, Canada

**Keywords:** Community-based screening, Prediabetes, Type 2 diabetes, Point-of-care testing, Health disparities, Underserved populations, Diabetes prevention

## Abstract

**Purpose of Review:**

This review mapped evidence on community-based screening interventions for early detection of prediabetes and type 2 diabetes (T2D), and identified barriers and strategies for developing and implementing such interventions in community settings for diverse populations.

**Recent Findings:**

Using the Arskey & O’Malley and Levac frameworks, we conducted a scoping review that identified 33 studies across 13 countries that developed and tested a community-based T2D screening intervention, utilizing risk assessment and Point-of-Care (POC) glucose testing. Screenings occurred in settings such as pharmacies (21%), faith-based centers (6%), and mobile vans (6%), with most studies from the United States (42%), Australia (16%), and Canada (9%). Post-screening, 89% of interventions offered referrals to primary care, while few connected participants to community programming. Barriers and strategies were mapped to the socioecological model to guide future development and implementation of early detection interventions in community settings.

**Summary:**

This review identified key factors for successful community-based T2D screening interventions, including adequate resources (i.e., funding and personnel), community engagement efforts, and accessible, feasible screening of T2D in community settings. POC testing proved valuable for early detection through immediate glucose results that would prompt potential interventions. However, challenges remain in ensuring long-term sustainability and feasibility of such approaches, as many interventions encountered high attrition rates due to challenges with referral pathways to health care and community programs, structural inequities, and lack of sustainable follow-up processes. Future research should focus on evaluating the cost-effectiveness and sustainable integration of these community-based T2D screening approaches into health systems for broader impact.

**Supplementary Information:**

The online version contains supplementary material available at 10.1007/s11892-025-01605-2.

## Introduction

The growing burden of type 2 diabetes (T2D) poses a significant challenge to health systems, economies, and the well-being of individuals and communities [1]. The rise in T2D cases can be attributed to socioeconomic inequalities, behavioural changes (i.e., reduced physical activity, unhealthy diets), aging and population shifts, environmental risks, and increasing obesity rates [2, 3]. If not detected and treated early, T2D can lead to severe complications such as neuropathy, nephropathy, and retinopathy, significantly reducing quality of life and life expectancy [4, 5]. Additionally, T2D increases the risk of lower extremity amputations, renal failure, blindness, and cardiovascular events, underscoring the severity and complexity of T2D-related complications [4–6]. Many people with T2D remain undiagnosed until complications arise, such as cardiovascular diseases (CVD), kidney disease, and blindness [7, 8]. As a result, undiagnosed T2D has significant implications for individuals, the health care system, and economies. Although early detection is vital to prevent future onset and potential complications, a major challenge remains with delayed or missed opportunities to identify high-risk populations living with prediabetes or undiagnosed T2D [2, 4, 5].

Although studies highlight the importance of early detection, there are many barriers such as limited access or lack of attachment to primary care, prolonged asymptomatic phases with elevated glucose levels (i.e., prediabetes), and missed opportunities to screen high-risk populations such as younger adults, or those from specific ethno-racial or socioeconomic groups who are at greater risk of developing T2D [7, 9]. Priority populations, defined as equity-seeking groups such as immigrants and low-income individuals, who face systemic barriers to equal access, opportunities, and resources due to factors such as race, ethnicity, gender, and socioeconomic status, encounter additional challenges [9–13]. These challenges include limited primary care access where screening often happens, difficulty in retaining continuity of care, discrimination, and a lack of culturally responsive care [9–13]. This limited access often increases reliance on emergency services and walk-in clinics, leading to inadequate follow-up and higher morbidity rates [9–13]. Addressing these gaps is critical, particularly in light of global healthcare shortages post-COVID-19, exacerbating existing challenges and further delaying the identification of high-risk populations [14, 15]. Thus, despite guidelines recommending more frequent screening for high-risk populations, many lack access to necessary services, creating a gap between recommended practices and real-world healthcare access [8–10, 16]. Hence, there is an urgent need for culturally sensitive, community-based interventions that offer accessible T2D screening services and connect individuals to primary care and community support [17–19]. These interventions being community-led and focusing on education, early detection, and prevention have the potential for reducing rising incidence of T2D and related complications [20].

Moreover, screening approaches outside of primary care, such as the CANRISK questionnaire and point-of-care (POC) devices for glycated hemoglobin (HbA1c), are valuable tools for identifying T2D [21, 22]. The CANRISK tool assesses risk factors like age, family history, and physical activity [21]. POC devices provide rapid testing of blood glucose levels without the need to send samples to a laboratory, making them more accessible and practical to use in community settings [21, 22]. Although, these screening approaches are becoming more available to use in various settings, there is currently limited evidence on the application of such tools in community settings for early detection of prediabetes and T2D combined with POC testing. Therefore, this scoping review aimed to better understand the components, approaches of community-based T2D screening interventions for early detection and to determine how these interventions were developed and tested across different settings and for diverse populations. A scoping review was necessary to map how such interventions were designed based on their characteristics, screening sites, detection tools, barrier encountered and strategies for addressing them, and the potential for providing valuable insight that will inform future community-led screening initiatives, particularly for priority populations. Therefore, the objectives of this scoping review were to: *1) identify community-based T2D screening interventions for priority populations; 2) examine approaches to conducting screening for detection of prediabetes and T2D *via* POC glucose testing and risk assessment questionnaires; and 3) identify barriers to and strategies for designing equitable, feasible and sustainable community-based T2D screening interventions.* The findings from this review will inform future community-based strategies for preventing T2D, particularly for priority populations.

## Methods

We conducted this scoping review following the Arksey and O’Malley methodology, which provides a six-stage foundational framework consisting of identifying the research question, identifying relevant studies, selecting the studies, charting the data, collating, summarizing, and reporting the results, and consulting with stakeholders to share and validate findings [23]. This approach was further enhanced by Levac et al.'s framework to support the consistency of how this review was conducted, emphasizing the importance of clearly defining the research purpose, using a step-by-step, team-based and iterative approach. This methodological rigour was applied in this review as the review progressed, and incorporated stakeholder consultation to increase the relevance and utility of the findings [24].

### Identifying Relevant Studies

A comprehensive search strategy was developed in collaboration with a University of Toronto librarian and validated through PRESS (Peer Review of Search Strategies). The strategy used five main concept headings: “population: equity-denied communities,” “type 2 diabetes or prediabetes,” “point-of-care devices,” “community-based interventions,” and “screening programs” (Table S1). Searches were conducted in five databases (OVID Medline, OVID EMBASE, OVID APA PsychINFO, Web of Science, and CINAHL) from December 12, 2023, to January 31, 2024. The search strategy incorporated controlled vocabulary (e.g., MeSH) where applicable (Table S1), and an example search from OVID Medline is shown in Supplemental Fig. 1.

A grey literature search followed from February 5 to 8, 2024, using a custom Google search (limited to the first 10 pages) and databases including the World Health Organization (WHO), Diabetes Canada, Public Health Agency of Canada (PHAC), and the Center for Disease Control (CDC). Grey literature searches often impose restrictions on search parameters such as word count and Boolean operators. Hence, these searches were adapted from the academic database search, focusing solely on the terms that best aligned with the research objectives (Table S1). Additionally, a manual review of the reference lists in the included studies was conducted for additional relevant articles.

To address peer reviewer recommendation of including studies up to January 2025, we conducted a secondary search using the updated time frame of January 2024 to January 2025. This additional search aimed to ensure the inclusion of the most recent evidence.

### Study Selection

The inclusion criteria were: 1) peer-reviewed studies (inclusive of primary and review studies) in English, 2) published from January 2000 to January 2025, 3) focused on undiagnosed prediabetes or T2D, 4) examining early detection interventions, and 5) using POC devices for detection. There were no geographic limitations.

Excluded studies were those focused on non-community settings (hospitals, clinics) unless screening was provided in community health centers and studies that did not use POC testing. Studies with no full text, focused on individuals already diagnosed with T2D or non-scientific publications were also excluded.

The search results were imported into Covidence, an online systematic review software that facilitates the review process, and duplicates were removed [25]. Two team members independently screened titles and abstracts, resolving conflicts through discussion to reach a consensus. Next, full texts were reviewed to confirm inclusion. Lastly, reference lists of the included studies were reviewed to identify and include any additional relevant studies.

**Fig. 1:**
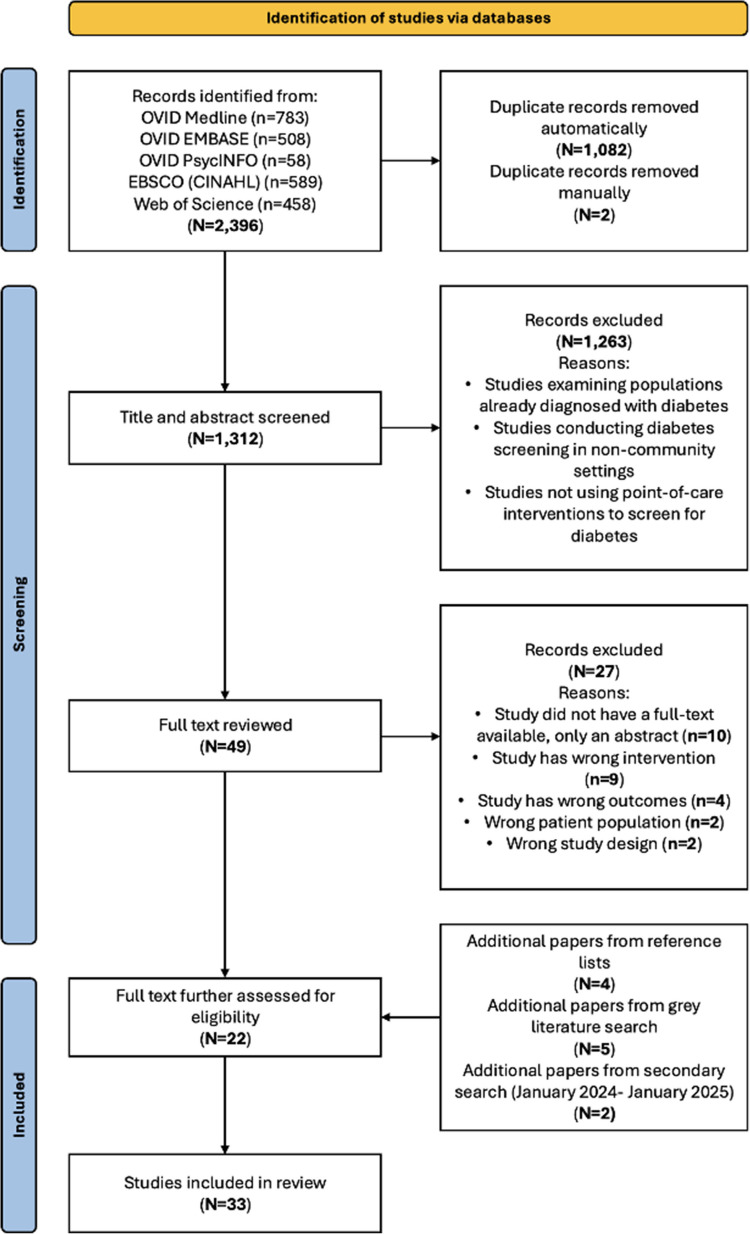
Preferred reporting items for systematic reviews and meta-analyses (PRISMA) flowchart

A total of 2,396 results were retrieved, with 1,312 unique references after removing 1,084 duplicates. The title and abstract screening narrowed the results to 49 studies, with 22 selected after a full-text review. An additional 11 studies (4 from reference lists, 5 from grey literature, 2 from the secondary search) brought the final total to 33 included studies. The review adhered to the PRISMA-ScR checklist (Fig. 1) [26].

### Charting the Data

Data extraction was performed using a template in Microsoft Excel version 16.78 by four members independently, where information from the 33 included studies was organized (Tables 1, 2, 3 and 4).

**Table 1:** Study population characteristics of included articles (*n* = 31) for prediabetes and type 2 diabetes community-based screening intervention

Author and date	Country	Sampl e Size	Population characteristics	Age range	Sex	Ethnic identity classification	Socio-economic position indicators	Health equity implications
Agarwal et al*.* [27]	Canada	526	Residents of Grimsby and surrounding areas	40 +	Male, Female	-	-	-
Alzubaidi et al. [28]	UAE	568	Arabic or English- speaking with no previous diagnosis of any diabetes, CVD, pregnancy, terminal illness, and/or severe mental illness	40–74	Male (53.35%), Female (46.65%)	Syrian, Pakistani, Indian, Egyptian, Jordanian and UAE citizens	Considered sociodemographic factors such as education (~ 25% had a high school diploma), over 33% reported having health insurance, and ~ 95% were born overseas and not UAE nationals	-
Andersen et al*.* [29]	Republic of the Marshall Islands	450	Self-identifying as Marshallese	18 +	Male (38.1%), Female (61.9%)	Marshallese resident	Accounted for historical trauma involving the Marshallese that shifted their diet tendency to one that is overly processed	Address health disparities and promote health equity among Marshallese adults through community-based participatory research (CBPR) and culturally sensitive approaches
Anyasodor et al*.* [30]	Nigeria	474	Adults residing in the Hospital catchment zones	18 +	Male (42.39%), Female (57.11%)	-	-	-
Bell et al*.* [31]	Portugal	494	Adults without any prior diabetes diagnosis, recruited from pharmacies	40 +	Male (41.3%), Female (58.7%)	Portuguese population	Self-reported educational level informed assumptions about health literacy levels	Pharmacists provided personalized counselling on healthy eating and physical activity to promote practical health knowledge, promoting health literacy, especially among those with lower educational attainment
Brennan et al*.* [32]	South Africa	1169	Participants had to be literate, be able to receive text messages, be suitable for nasopharyngeal sample collection, not deemed vulnerable by study personnel, not at risk of protocol non- compliance, and not diagnosed positive for COVID-19 within past 3 months	18 +	Male (58.3%), Female (41.6%)	-	-	-
Camargo et al*.* [33]	Brazil	542	Individuals in rural areas covered by family health units	18 +	Male (33.6%), Female (66.4%)	White, Brown, Black, and Other	Accounted for average monthly income (US$) for both family and personal income	Improve access to Glycated Hemoglobin tests in underserved rural communities
Davidson et al*.* [34]	USA	1542	Individuals without history of any diabetes, who exhibited 1 + risk factors for T2D	40–93	Male (34.7%), Female (65.3%)	African American and Latino populations in the United States	-	Address high prevalence of diabetes in African American/Latino populations and disparities in primary care access
Genco et al*.* [35]	USA	1022	Dental patients who were not aware of their diabetic status	45–90	Male (38.8%), Female (61.2%)	-	-	-
Glurich et al*.* [36]	USA	127	No pre-existing diagnosis of T2D/prediabetes, documentation of known risk factors for diabetes	-	Male (35.4%), Female (64.6%)	White race or Hispanic	-	-
González and Jarque, [37]	Spain	434	Individuals that visited community pharmacies and no prior diagnosis of any diabetes	18 +	Male (40%), Female (60%)	-	-	-
Gracey et al*.* [38]	Australia	416	Aboriginal individuals living in remote Aboriginal communities	Mentions pre-adult (younger than 18) and adults (older than 18)	Male, Female	Australian Aboriginal	Population lacked information about the connections between nutrition, exercise, and health, access to fresh, affordable, healthy food at local stores	Highlights the importance of empowering individuals, promoting health equity, and addressing systemic barriers to better health
Hadenfeldt et al*.* [39]	USA	478	Homeless adults participating in the annual 1-day Project Homeless Connect Omaha (PHCO), determined to be at risk for developing T2D	26–82	Male (68%), Female (29%)	White (not Hispanic/Latino), Black or African American, White (Hispanic/Latino), Native Americans	Identified adults who are unhoused	Target adults experiencing homelessness, a group that faces significant challenges in accessing healthcare and managing chronic conditions
Harris et al*.* [40]	USA	2451	Rural residents	-	Male, Female	-	Rural populations in Maine, that are often considered to have high vulnerability to cardiovascular disease	Address health disparities and promoting equitable access to healthcare services to those in rural regions through community-based screening programs that do not rely solely on primary care physicians
Herman et al*.* [41]	USA	1033	Adults with no history of diabetes who were being seen for routine checkups and cleanings	30 +	Male, Female	-	-	-
Kerkhoff et al*.* [42]	USA	923	> 80% were Latinx self- identifying, and had household annual incomes < 50 000 USD, < 50% did not have health insurance, > 50% did not have access to primary care clinician	18 +	Male (44.3%), Female (55%)	Latinx, African American/Black, American Indian or Alaska Native, Asian, Pacific Islander or Native Hawaiian, White, other (not identifying with other categories)	Included low-income Latino persons not engaged in formal health care services	Build trust to overcome historical medical mistrust, and structural barriers to accessing primary care as well as immigration concerns of underserved communities
Kim et al*.* [43]	USA	285	Adult individuals visiting a Federally Qualified Health Center (FQHC)	18–75	Male (43.9%), Female (56.1%)	-	Patient populations at FQHCs, that are often considered to have more complex health concerns (i.e. related to socioeconomic issues)	Prioritize enhancing screening in underserved populations, and recognize the social determinants of health on underserved populations
Krass et al*.* [44]	Australia	2242	Consenting screening participants who had been referred to their GP, with a 20% random sample of non- referred screened participants	35–74	Male (44.5%), Female (55.5%)	-	-	-
McElfish et al*.* [45]	USA	378	Marshallese adults (Pacific Islanders) with a BMI of 25 kg/m^2^ or higher	18 +	Male (43.4%), Female (56.6%)	Marshallese individuals	Identified that in past year, 40.3% of participants couldn't see a doctor due to costs, while 42.5% had no health insurance, and uninsured participants were over twice as likely to be undiagnosed	Highlights the barriers that Marshallese migrants'excluded from federal healthcare programs (Medicaid) experience
Millard et al*.* [46]	USA	2,332	At high risk for T2D or prediabetes and not pregnant	≥ 25	Male (46%), Female (54%)	Hispanic	Considered low-income backgrounds from medically isolated communities at high risk for T2D or prediabetes	Incorporate culturally tailored elements, engaging the community, and eliminating barriers to access
Misra et al*.* [47]	USA	538	Not pregnant, residents of the rural communities being screened	18 +	Male (30.9%), Female (69.1%)	Non-Hispanic Whites and minorities	-	Identify and address the high rates of diabetes within West Virginia, and those that are hard to reach or without routine medical access in rural areas
Osorio et al*.* [48]	USA	290	English-speaking black men without a history of any diabetes	18 +	Male	Black individuals (including those who specifically identified as Caribbean, West Indies origin or foreign born)	Targeted neighborhoods identified as having high prevalence of poor glycemic control	Address the challenges and socioeconomic barriers that Black men living in urban areas face to achieve good health (i.e., poor food environments and difficulty in obtaining primary care)
Ralph- Campbell et al*.* [49]	Canada	693	Adult Métis	25 +	Male (39%), Female (61%)	Métis	Métis populations have challenges in accessing healthcare, indicating a potential barrier to health services	Address emerging diabetes epidemic within the Métis population and recognize their barriers to healthcare access
Risøy et al*.* [50]	Norway	211	Norwegian or English- speaking pharmacy customers	18 +	Male (37%), Female (63%)	European and non- European	-	-
Risøy et al*.* [51]	Norway	245	Interested pharmacy customers	≥ 45	Male (25%), Female (67%)	Western or non- Western (origin is from Asia or Africa)	-	-
Rowan et al*.* [52]	Canada	670	Able to undergo a capillary blood test	18 +	Male (29%), Female (71%)	High-risk communities (South Asian, African- Caribbean, Chinese and Aboriginal descent)	-	Focus on specific ethnic groups known to be at elevated risk for developing T2D
Serrano et al*.* [53]	Australia	510	Voluntary adults	-	-	Aboriginal/Torres Strait Islander, European/Caucasian, Asian, Indian/Subcontinent, Middle Eastern, Pacific Islands	-	Focus on specific ethnic groups known to be at elevated risk for developing T2D
Shemesh et al*.* [54]	Australia	39	Individuals from remote Aboriginal communities	18–71	-	Australian Aboriginal	-	Identified need for addressing health disparities and promoting health equity to those in rural regions through reliable and portable point-of-care instruments
Shephard et al*.* [55]	Australia	380	Voluntary Aboriginal adults	15–83	Male (30-47%), Female (53–70%)	Australian Aboriginal	Geographic isolation, limited employment opportunities, low income and poor access to housing and education	
Tabaei et al*.* [56]	USA	3,506	Voluntary adults	Minimum age not specified, mentions > 20 and < 20	Male (37%), Female (63%)	African Americans, Native Americans, and White Americans	-	Calls for efforts to target medically underserved individuals who may not have access to routine health care, lack insurance, or do not have a primary care physician
Tantipoj et al*.* [57]	Thailand	724	Dental patients with no history of hyperglycemia, without severe anemia, polycythemia, or conditions causing secondary diabetes mellitus, able to complete demographic forms	25 +	Male (22.6%), Female (77.5%)	-	-	-
Wensil et al*.* [58]	USA	206	Residents in migrant camps able to provide informed consent, with no previous diabetes diagnoses, not pregnant, and not treated with any antihyperglycemic agents	18 +	Male (92.7%), Female (7.3%)	Hispanic identifying except for one participant	Patient population as migrant farm workers are often considered underserved/vulnerable due to factors such as low income, limited access to healthcare, language barriers	Focus on the vulnerable nature of migrant farm worker populations and their increased risk for undiagnosed T2DM/prediabetes cases and challenge of managing chronic diseases
Yonel et al*.* [59]	UK	101	Consent to trial participation	40 +	Male (47-53%), Female (47–53%)	White/Caucasian identifying except for one participant	-	-

**Table 2:** Prediabetes and type 2 diabetes community-based screening intervention characteristics

Author and date	Recruitment methods		Screening site	Screening Personnel	Biomarker measures	Point-of-care (POC) device used
Agarwal et al*.* [27]	Participants were recruited via local media, (on the newspaper, radio, and TV), family physicians mailed invitations to patients 40 + without any diabetes		Pharmacies	Locally trained volunteers	Fasting blood glucose and HbA1C	-
Alzubaidi et al. [28]	Pharmacists directly recruited participants during visits, supplemented by pharmacy-based posters/flyers and social media		Pharmacies	Pharmacists	BMI, HbA1c	Roche Cobas b 101 dual system
Andersen et al*.* [29]	Community members recruited churches via radio, text messaging, and invitations, including non-participating churches interested in joining		Church setting	Study team	HbA1c, BMI, height, and blood pressure	A1CNow + test
Anyasodor et al*.* [30]	Recruitment involved awareness lectures held in marketplaces, schools, churches, and hospitals within Ndokwa communities		Communities and a dental clinic	Study team	Fasting blood glucose (FBG), random blood glucose, lipid profiles	CardioChek PA analyzer
Bell et al*.* [31]	Convenience sampling from community pharmacy visitors		Pharmacies	Pharmacists	HbA1c, random capillary blood glucose, fasting capillary blood glucose	Roche Cobas b 101 system
Brennan et al*.* [32]	Approaching commuters, vendors, and drivers at the Germiston taxi rank		Busy public transport hub	Study team	Random blood glucose, BMI, blood pressure	Glucose meter
Camargo et al*.* [33]	-		Basic Health Unit (primary care clinic) and routine activities, like patient fairs	Nurses and physicians	Blood pressure, HbA1c, capillary blood glucose, body mass index (BMI)	Abbott Affinion™ 2 Analyzer
Davidson et al*.* [34]	Outreach at churches, health fairs, community events, clinics, and through flyers		Churches, community health fairs, senior citizen sites, and clinics	Study team	HbA1c levels, fasting glucose, and blood pressure	A1C Now +
Genco et al*.* [35]	All staff members were informed and verbally encouraged to recruit patients		General and periodontal specialty dental offices and community dental clinic	Clinical coordinators	HbA1c	A1C Now +
Glurich et al*.* [36]	-		Dental clinics	Dental providers (dentists and hygienist)	HbA1c, fasting and random blood glucose, blood pressure, body mass index (BMI)	DCA Vantage HbA1c Analyzer
González and Jarque, [37]	-		Pharmacies	Pharmacists	Fasting glucose (blood glucose) and HbA1C	Glucomen® Aereo 2 K from A. MENARINI diagnostics
Gracey et al*.* [38]	Outreach to the communities by trusted leaders (UFPA members/staff) in public community meetings		-	Local clinical staff/Aboriginal health workers	Blood pressure, HbA1c, fasting glucose, and BMI	DCA2000 + analyzer
Hadenfeldt et al*.* [39]	Participants recruited at Project Homeless Connect Omaha (PHCO) outreach event		Health and social services outreach event	Health professional students and faculty	Hb1C	-
Harris et al*.* [40]		Mobile van offered free voluntary screenings at community and workplace events, including agricultural fairs	Mobile vans	Nurse practitioner	Random blood glucose, blood pressure	Accu-Chek, ‘compact’ model
Herman et al*.* [41]		-	Dental practices and Research Unit (for the HbA1c testing)	Study team (research assistants)	BMI, random blood glucose, HbA1c	FreeStyle Lite blood glucose meter (for random blood glucose), Tosoh G7 HPLC Analyzer (for HbA1c)
Kerkhoff et al*.* [42]		Participants recruited through word-of-mouth, text notifications, COVID-19 vaccine visits, flyers, social media, news outlets, or passing by the site	Outdoor, neighborhood- based setting near busy transportation hubs	Laboratory assistant	HbA1c	-
Kim et al*.* [43]		-	Primary care clinic at the FQHC	Medical Assistants	HbA1C	-
Krass et al*.* [44]		-	Pharmacies	Pharmacists	Fasting and random blood glucose, HbA1c	-
McElfish et al*.* [45]		Outreach conducted in community settings, including churches, with consent from bilingual Marshallese study staff	Church setting	Study team	BMI, blood pressure, HbA1C	Rapid A1C test kit and DCA Vantage HbA1c Analyzer
Millard et al*.* [46]		Recruitment methods included word-of-mouth, a sugar content exhibit, and free diabetes tests/screenings	Flea market community setting	Study team	HbA1c	DCA Vantage HbA1c Analyzer
Misra et al*.* [47]		Advertisement for screenings included free A1c testing during healthcare community event	Health fairs, parent/teacher nights, basketball games, library events	Study team (extension agents)	HbA1c	A1C Now +
Osorio et al*.* [48]		Customers approached in a barbershop; some initially declined but agreed after encouragement from their barber	Barbershop owned by black individuals	Study team	HbA1c	A1CNow + test
Ralph-Campbell et al*.* [49]		-	Mobile clinics	Health professionals	HbA1C, fasting glucose, blood pressure, and BMI	DCA2000 + analyzer
Risøy et al*.* [50]		Pharmacists informed customers via leaflets, newspapers, social media, and word-of-mouth, receiving 100 NOK per recruited participant	Pharmacies	Pharmacists	HbA1c	DCA Vantage HbA1c Analyzer
Risøy et al*.* [51]		Participation was free for pharmacy customers, with pharmacies receiving approximately €10 per recruited participant as incentives	Pharmacies	Pharmacists	HbA1c	DCA Vantage HbA1c Analyzer
Rowan et al*.* [52]		Participants recruited via community partnerships, printed materials, email lists, and public screenings in high-traffic areas like malls and health centers	Community health centers, shopping malls	Study team	HbA1c, BMI	Bio-Rad in2it
Serrano et al*.* [53]		Community outreach (i.e., on-site advertising, community announcements, social media posts) through local organizations and cultural events	Community-based events (i.e., Tamil Arts and Culture Festival, NAIDOC Week, Blacktown Worker’s Club)	Nurses	HbA1c	Abbott Affinion™ 2 Analyzer
Shemesh et al*.* [54]		-	-	Study team	HbA1c, fasting glucose	DCA2000 + analyzer
Shephard et al*.* [55]		Culturally appropriate information sheets were developed and given out in meetings with local Aboriginal community members	Ecotourism center, college, women’s center, community fair, mobile van	Trained Aboriginal health workers	Fasting glucose, HbA1C, urine ACR, cholesterol	DCA2000 + analyzer
Tabaei et al*.* [56]		Screening was performed as part of community awareness and outreach programs, and comprehensive work site wellness programs	Hospitals, health fairs, shopping centres, work sites, community centers	Study team	Fasting and random blood glucose	-
Tantipoj et al*.* [57]		Approaching those that were seeking dental treatment already	Community dental clinic and mobile dental service	-	BMI, blood pressure, HbA1c	DCA Vantage HbA1c Analyzer
Wensil et al*.* [58]		-	Migrant camps	Study team	HbA1C, random plasma glucose	A1C Now +
Yonel et al*.* [59]		Patients over 40 attending the dental practice for NHS services were approached until target numbers were met	Dental practice, pharmacies	Study team	HbA1c, blood pressure readings, BMI	DCA Vantage HbA1c Analyzer

**Table 3:** Prediabetes and Type 2 diabetes community-based screening intervention process and referral pathways

Author and date	Study duration	Screening procedures	Population screened	Percenta ge that met criteria for type 2 diabetes	Percenta ge that met criteria for pre- diabetes	Referral pathways post-screening	Evidence of community programing post- screening
Agarwal et al*.* [27]	2 months	Questionnaire (FINDRISC and Cambridge Diabetes risk assessment questionnaire) + POCT	No specific population	-	-	Follow ups were provided (invited to counselling session), with a copy of results sent to family physicians	-
Alzubaidi et al. [28]	-	Questionnaire (American Diabetes Association (ADA) diabetes risk) + POCT	Pharmacy customers	12%	26%	At-risk participants received a referral letter summarizing screening results, advising a physician visit within four weeks	-
Andersen et al*.* [29]	-	Questionnaire (adapted from the Behavioral Risk Factor Surveillance System) + POCT	Underserved ethnic communities	45.30%	17.80%	Individuals with high HbA1c levels were scheduled appointments with a health care provider	-
Anyasodor et al*.*, [30]	4 months	Questionnaire (WHO questionnaire) + POCT	No specific population	18%	38.80%	-	-
Bell et al*.* [31]	6 months and 5 months	Questionnaire (FINDRISC) + POCT	Pharmacy customers	8.7%	29.8%	Participants with HbA1c > 5.7% referred to GP	-
Brennan et al*.* [32]	2 months	POCT	No specific population	5.20%	-	Referring to primary care those with abnormal results	-
Camargo et al*.* [33]	6 months	Questionnaire + POCT	Rural residents	3%	5.72%	Individuals with high HbA1c levels were asked to repeat the test within 3 months at the primary care unit, if HbA1c still high, they were referred to physician	-
Davidson et al*.* [34]	-	POCT	Underserved ethnic communities	25%	40%	-	-
Genco et al*.* [35]	-	Questionnaire (American Diabetes Association Diabetes Risk Test) + POCT	Dental patients	-	40.70%	Referring participants with abnormal results to a physician for diagnostic testing	-
Glurich et al*.* [36]	11 months	Questionnaire (intake screening questionnaire, demographic and comorbidity profile questionnaire, American Diabetes Association Diabetes Risk Test) + POCT	Dental patients	5.51%	43.30%	Received telephonic follow-up 3 months following HbA1c screening to determine compliance with recommended triage to medical providers for further monitoring	-
González and Jarque [37]	20 days	Questionnaire (FINDRISC) + POCT	Pharmacy customers	-	20.50%	Referrals to physicians were made for those with abnormal results	-
Gracey et al*.* [38]	Range from many months - 3 years (in different communities)	Questionnaire + POCT	Underserved ethnic communities, rural residents	-	-	Referring to clinical services to those with abnormal health indicators or risk factors	Yes, lifestyle modification programs with continued education and support
Hadenfeldt et al*.* [39]	1 day	Questionnaire ("Are You at Risk for Developing Type 2 Diabetes?"developed by the American Diabetes Association) + POCT	Unhoused individuals	4%	32%	Referred participants with abnormal results to federally funded free clinic	-
Harris et al*.* [40]	4 years	POCT	Rural residents	4.40%	10.70%	Advised to contact primary health care provider	-
Herman et al*.*, 2015 [41]	20 months	Questionnaire + POCT	Dental patients	1.30%	28.70%	Referring to primary care those with abnormal results	-
Kerkhoff et al*.* [42]	2 months	POCT	Underserved ethnic communities	33.90%	12.20%	Uninsured individuals with diabetes were referred to a community health clinic for a new patient appointment. Those with insurance but out of care received coaching from a CHW to reengage in care	-
Kim et al*.* [43]	2 months	Questionnaire (Prediabetes Risk Test) + POCT	Healthcare centre visitors	-	7%	Patients identified with T2D had results and management discussed by healthcare providers	-
Krass et al*.* [44]	-	Questionnaire (AUSDRISK) + POCT	Pharmacy customers	-	-	Referring participants with abnormal results to their GP	-
McElfish et al [45]	2 years	Questionnaire + POCT	Underserved ethnic communities	48.20%	-	Follow ups and further assessments were available for those with diagnosis (referred to primary care)	-
Millard et al*.* [46]	26 months	Health education + POCT	Underserved ethnic communities	25.30%	28.50%	Referring participants with abnormal results to their GP	-
Misra et al*.* [47]	1 year	Questionnaire (CDC Risk Calculator) + POCT	Rural residents	-	61.80%	Participants with abnormal results were referred to a healthcare provider and encouraged to participate in community programming	Yes, community programs such as"Dining with Diabetes"and other T2D education and lifestyle programs available in community
Osorio et al*.* [48]	17 months	POCT	Underserved ethnic communities	10%	28.30%	Participants with abnormal results were counseled on diet and physical activity modifications, and given contact information for local primary care clinics	-
Ralph-Campbell et al*.* [49]	3.5 years	POCT	Underserved ethnic communities	5.30%	20.30%	Refer to the existing health system for management and follow-up care	-
Risøy et al*.* [50]	2 months	Questionnaire (European backgrounds filled out FINDRISC, while non-European backgrounds filled out Diabetes UK-test) + POCT + follow-up Questionnaire	Pharmacy customers	1.42%	5.69%	Advising participants with abnormal results to visit their GP	-
Risøy et al*.* [51]	8 months	Questionnaire (FINDRISC for those with Western backgrounds, Leicester Risk Assessment (LRA) for those with non-Western backgrounds) + POCT	Pharmacy customers	9%	43%	If HbA1c indicated pre-diabetes, they were recommended to visit their GP within the year, if HbA1c indicated diabetes they were asked to visit GP as soon as possible	-
Rowan et al*.* [52]	-	Questionnaire (FINDRISC and CANRISK modified to make the PRE-PAID Questionnaire) + POCT	Underserved ethnic communities	-	-	-	-
Serrano et al*.* [53]	6 months	POCT	Underserved ethnic communities	19%	38%	Participants with HbA1c > 6.4% advised to consult GP, provided list of local healthcare providers for those without a GP, referral to local health services (i.e., optometrists and podiatrists), linked to ongoing lifestyle modification programs	Yes, participants were offered enrollment in lifestyle modification programs and received a local lifestyle education pack (including access to free or low-cost exercise classes)
Shemesh et al*.* [54]	-	POCT	Underserved ethnic communities, rural residents	-	-	-	-
Shephard et al*.* [55]	-	POCT	Underserved ethnic communities	15–18%	-	Referral to doctor for those with abnormal results	-
Tabaei et al*.* [56]	7 months	Questionnaire ("Take the Test. Know the Score"from the American Diabetes Association) + POCT	Underserved ethnic communities	0.50%	-	Follow ups and further assessments were available for those with abnormal results (most had primary care physicians)	-
Tantipoj et al*.* [57]	-	Questionnaire + periodontal examination + POCT	Dental patients	5.60%	28.20%	Advised those with prediabetes to make lifestyle adjustments and repeat the blood test annually. Subjects identified as having T2D were referred to their physician	-
Wensil et al*.* [58]	3 months	Questionnaire + POCT	Underserved ethnic communities	-	-	Referred for laboratory testing, and then results reviewed by a primary healthcare provider	-
Yonel et al*.* [59]	8–14 days	Questionnaire (“Diabetes Risk Score” developed by Leicester University and Diabetes UK) + POCT	Dental patients, pharmacy customers	4.40%	47%	Referring participants with abnormal results to their GP	-

**Table 4:** Challenges, strategies and recommendations in prediabetes and type 2 diabetes community-based screening interventions

Author and date	Challenges that presented barriers to community-based screening	Strategies and approaches that supported community-based screening	Recommendations for future design and implementation of community-based screening interventions
Agarwal et al*.* [27]	• The program heavily relied on volunteers, leading to challenges in crowded and busy venues for assessments • Some attendees did not fast beforehand, potentially affecting accuracy of results • The participants were generally more educated, health-conscious, and less likely to develop diabetes, posing difficulties in reaching a diverse participant base through outreach efforts	• Participants found pre-attendance fasting easy • Majority reported the blood test as not painful • Found the clinic location convenient, found the program informative, and appreciated helpful staff • Volunteer participation was beneficial, keeping costs low and reducing the need for health professionals, positively impacting participants	• Targeting individuals not reached by advertisements, particularly those with lower education levels, to mitigate selection bias
Alzubaidi et al. [28]	• Direct pharmacist-to-physician referral was not possible due to limitations within the healthcare system	• Referral pathway did not have direct pharmacist involvement in physician referral, the referral was only to the screened individual, but it remained effective • The appeal of point-of-care testing devices to screened participants was notable, perceived as more"legitimate"and encouraged follow-up	• Exploring pharmacy-based screening models in the country with effective direct referral pathways involving physicians in primary care and providing individual support after positive screening is recommended
Andersen et al*.* [29]	• Previous research led to distrust between population and researchers • Supply chain issues caused delays, with unreliable tracking data and supplies taking over a month to arrive • Maintaining correct test kit temperatures was challenging in churches without air conditioning • Church setting was difficult as the building needed to be unlocked or other events were taking place	• Study employed a community-based participatory research (CBPR) approach due to past trauma experienced by the Marshallese people • Community advisory board included RMI community partners to lead the assessment • Questionnaire length was reduced from prior studies to ensure accurate responses and reduce participant burden	• To improve supply chain management, strategies should be developed to ensure proper temperature maintenance of test kits • Community engagement efforts should be enhanced to address challenges related to event scheduling and venue availability • Recognizing limitations of measurement tools like BMI, alternative approaches to assess weight- related risk factors in a culturally sensitive manner should be explored
Anyasodor et al*.* [30]	-	• By utilizing orodental visits for screening, individuals could be screened for during routine dental appointments, improving detection while reducing participant burden	• Future research in Nigeria could benefit from addressing socio-economic disparities, health equity, and social determinants of health concerning diabetes screening and orodental health • Exploring the effectiveness and cost-effectiveness of opportunistic screening strategies and community engagement programs to promote diabetes screening and oral health
Bell et al*.* [31]	• Limited health literacy among participants, especially those with lower educational levels • Lack of ongoing support after the initial screening, as the study did not link participants to long-term community programs, limiting sustained lifestyle changes	• Pharmacy-based screening was accessible and cost-effective (reduced barriers to transportation and scheduling) • Provided immediate lifestyle counseling after screening	• Increase health literacy interventions at community pharmacies • Further integrate screening with healthcare networks to ensure follow-up care • Emphasize the role of community pharmacists in educating patients on diabetes prevention, particularly in high-risk groups
Brennan et al*.* [32]	• Screening at commuting centers presents challenges in linking individuals to care compared to diagnoses made at healthcare facilities • Single blood glucose or blood pressure measurement is insufficient for diagnosing diabetes	• The screening intervention could be seamlessly integrated into existing mobile testing efforts led by health staff, such as those targeting HIV and tuberculosis	• Recruitment bias may exist, given that taxi ranks primarily serve employed individuals commuting to or from work, so more males than females were enrolled, reflective of employment trends in South Africa
Camargo et al*.* [33]	• Limited awareness of screening importance, and cultural beliefs • Logistical hurdles in reaching remote areas. • Limited access to healthcare information • Low health literacy• Competing daily priorities	• Integrating HbA1c testing into routine activities (i.e. health fairs and patient group visits) that are integrated into the activities of the family health team, increased new diagnosis rates	• Expanding HbA1c testing to more rural communities and healthcare settings requires developing and implementing community engagement strategies to increase diabetes awareness, promote regular screenings, and encourage active participation
Davidson et al*.* [34]	• Difficult to obtain informed consent for screening HbA1c tests conducted at community events and ensuring proper follow-up for participants with positive results • Difficult to adapt to changing diagnostic criteria for prediabetes	• Utilized easily identifiable risk factors like central obesity, hypertension, and positive family history to streamline targeting high-risk individuals for screening	• Community-based screenings hold promise as a means of addressing the healthcare needs of high- risk populations, as well as utilizing easily identifiable risk factors to target high-risk populations
Genco et al*.* [35]	• Patients'resistance to follow-up (may stem from denial, optimism bias, fear of receiving a diabetes diagnosis, financial concerns, lack of motivation in asymptomatic individuals, limited access to medical care, and other health behavior-related factors)	• Implementing more formal contracts with patients to ensure follow-up with referrals or increasing the utilization of auxiliaries or community health workers to provide support and monitor follow-up	• Importance of evaluating and addressing the reasons for poor follow-up with referrals for diagnostic assessment • Advocates for further research into potential impact of expanded insurance coverage in incentivizing patients to seek a diagnosis after screening • Recommends conducting a cost–benefit analysis of screening for undiagnosed diabetes in dental offices
Glurich et al*.* [36]	• HbA1c screening was not feasible for all patients, as some sought treatment for dental emergencies	• 90% of the patients were compliant with follow-up recommendations at the 3-month mark after screening	• Need to expand and promote interdisciplinary efforts across primary dental and medical settings, focusing on larger sample sizes to identify and manage high-risk individuals • Further research to explore cost-effectiveness • Need for community engagement, patient education, and awareness campaigns to enhance the reach and impact of glycemic screening programs in dental settings
González and Jarque [37]	• The return rate from patients referred to physicians was low (< 30%) in urban areas, contrasting with a higher rate in rural areas (85.2%), suggesting a higher level of trust in rural pharmacists, leading to differences in patient engagement and follow-up	• Community pharmacies were effective in diabetes screening, with a 3% detection rate of newly diagnosed patients	• Exploration into methods to increase return rate of patients referred to GPs after abnormal results in urban populations
Gracey et al*.* [38]	• Limited Aboriginal trust in mainstream health services • Individuals with other prioritized commitments (i.e. funerals and ceremonies) • Remote location of communities and geographic obstacles like seasonal weather affecting travel • Limited funding, infrastructure, and resources for health programs • Scarcity of healthcare professionals	• Implementing a community-driven approach involving local partnerships and community engagement (includes culturally appropriate health promotion, training, and capacity building within the community to take ownership of health initiatives) • Leveraging existing resources, partnerships, and community support to optimize the use of available funding and minimize operational costs	• Collaborating with sectors like education, housing, and social services can address broader social determinants of health • Robust data collection and monitoring mechanisms is essential for tracking progress, evaluating outcomes, and identifying areas for improvement • Continuing to train local community members
Hadenfeldt et al*.* [39]	• The study relied on volunteer health professions students to interpret the risk assessment form, conduct POC test, and provide education	• 1-day fair targeting a specific population	• Given that it was a one-day fair, caution should be exercised when generalizing the findings to other settings
Harris et al*.* [40]	• Geographic challenges in accessing rural areas • Detailed demographic collection was lacking • Incomplete attendance data impedes calculation of acceptance rates • Limited follow-up (i.e. only one phone call)	-	• Expand screening to more rural areas • Complete attendance and demographic data should be collected • Continue education on the importance of follow-up care
Herman et al*.* [41]	• Low response rates for definitive diagnostic testing • Participant concerns regarding finger prick blood collection • Barriers to widespread implementation in dental offices (i.e. reimbursement issues)	• The questionnaire effectively identifies individuals at high risk	• Future research should look into methods to recruit a more diverse population and strengthen follow- up care in those referred
Kerkhoff et al*.* [42]	• Reaching individuals who had not previously been tested for any diabetes and were not engaged in formal healthcare services	• Clients felt comfortable getting screened at the location due to its convenient, highly visible outdoors neighborhood setting, free services, and recommendations from trusted friends, colleagues, or family members • Site was open on weekends and allowed community members to drop-in for free testing without appointments or ID requirements • Trained staff, including Spanish speakers, contributed to welcoming atmosphere • Availability of multi-disease testing further encouraged participation, with individuals testing for COVID-19 also screened for diabetes	• The study leveraged trust from a COVID-19 testing site, which took 15 months to build with the community, potentially limiting generalizability to sites lacking this pre-established trust • Further research on optimal strategies to overcome multilevel barriers to care linkage following new diabetes diagnoses in community-based testing programs is urgently needed
Kim et al*.* [43]	• Data capture within the electronic health record (EHR) system faced challenges due to inadequate IT support • Use of paper based and manually documented Prediabetes Risk Test (PRT) resulted in reduced participation and incomplete data collection	• Offering additional EHR training and IT support to facilitate real-time problem-solving	• Transition to a digital format for the PRT with automatic documentation in the EHR system to streamline screening and enhance data collection
Krass et al*.* [44]	• Approximately 10% of participants expressed dissatisfaction with the information provided or were disappointed by the lack of an actual blood test at the pharmacy• Small number felt that pharmacists should not be responsible for screening, with preference for general practitioners to fulfill this role• Some individuals insisted on point-of-care testing regardless of their risk score	• Over 95% of respondents expressed satisfaction with the pharmacist's professional knowledge and explanation of screening results, while over 90% indicated they would recommend the service• More than 50% reported making lifestyle changes post-attendance	• Consumer surveys indicated that screening services lacking a finger-prick test were perceived as less valuable by both consumers and pharmacists, potentially undermining confidence in the results• Establishing an effective communication channel between GPs and pharmacists would be essential for facilitating continuity of care
McElfish et al*.* [45]	• Self-reported questionnaire introduces potential bias • Absence of control or comparator groups limits population comparisons	• Recruitment from community settings like churches and bilingual Marshallese staff fostered trust and rapport with the Marshallese community	• Future studies should incorporate longitudinal designs to monitor health outcomes over time and evaluate intervention effectiveness • Future research should include control groups, such as non-Marshallese, to offer insights into unique health challenges within Marshallese population
Millard et al*.* [46]	• Brief encounters (10 min) posed challenges in effectively transmitting information about improving eating habits and physical activity, particularly overcoming language barriers and limited health literacy	• Regular weekly screening schedule for reliable access • Innovative recruitment techniques, like displaying sugar content, to attract participants • Brief health education provided at the site • Culturally appropriate health education materials developed • Height and weight measured in private for cultural sensitivity • Free screening, tests, and clinic appointments provided • Family members engaged for social support • Welcoming environment with friendly staff • User-friendly project layout designed for accessibility	• Implement extended follow-up strategies • Develop culturally appropriate educational materials • Explore efficient clinic care for diabetes • Provide additional training for healthcare providers on nutrition and lifestyle interventions
Misra et al*.* [47]	• Limited participation from minority adults in screening • Self-reported information on questionnaires could be influenced by social desirability bias, potentially underestimating their risk • Site locations may also attract individuals with higher education levels or greater health concerns	• Screenings were strategically scheduled on weekdays and weekends, including existing programming/events, to increase participation • Extension agents, trusted in the community, played a key role in facilitating screenings	• Community events yielded a stronger response for diabetes screenings compared to health fairs, but the sample may skew towards higher-educated participants • Given high obesity rates and limited access to medical care, a non-fasting universal screening method could be more reasonable and accessible
Osorio et al*.* [48]	• Lack of time or interest, fear of knowing the result, or fear of needles for not wanting testing • Only one individual declined testing specifically because the setting was a barbershop	• Approximately one-third of men approached in barbershops owned by black individuals were willing to be screened, there was a perceived communicated sense of trust	• A single test result was used to consider a diagnosis of diabetes; however, confirmatory testing was not performed in the study and should be considered in future research • The study sample and participation rate may not be representative of other barbershops
Ralph-Campbell et al*.* [49]	• Underreporting in census data may lead to potential underestimation of T2D/prediabetes prevalence rates	• Mobile vans with health professionals and portable diagnostic equipment were beneficial in remote environment	• The use of HbA1c testing and POC can be beneficial in remote and more inaccessible locations for screening
Risøy et al*.* [50]	• Low awareness among customers • Media discrepancy affecting recruitment rates • Time-consuming follow-up • Incomplete email addresses causing delivery issues • Training misunderstandings • Screening process affecting pharmacy operations • Low participation among non-European populations	• Multifaceted outreach (i.e. social media, news) • Tailored risk assessment tools • Immediate HbA1c results • Free service • Recruitment incentives for pharmacies • Efficient use of project funding	• Consider offering risk assessments and promotional material in more diverse languages (i.e. Arabic and Somali) to enhance participation diversity • Ensure ongoing quality assurance for HbA1c measurements through participation in external control programs • Develop clear referral pathways for high-risk participants, including guidance on follow-up care and lifestyle interventions • Implement follow-up strategies to ensure timely feedback and encourage adherence to recommended actions, monitoring progress over time
Risøy et al*.* [51]	• Recruitment of pharmacies and participants proved challenging, with less than 3% of invited pharmacies participating in the study	• Training sessions were conducted with pharmacy staff on utilizing POCT and conveying information to participants	• More active recruitment strategy and extended recruitment period needed to boost uptake, particularly targeting specific populations like non- Western immigrants at higher risk • Enhancing communication between pharmacies and GPs could improve follow-up and continuity of care for high-risk individuals • Larger studies needed to assess cost-effectiveness of diabetes risk assessment services
Rowan et al*.* [52]	• Cultural differences, language barriers, and awareness challenges • Expenses for personnel training, equipment, analyses, incentives, securing funding, recruiting culturally competent staff • Identifying suitable locations in high-risk ethnic communities, considering accessibility, trustworthiness, and cultural appropriateness	• Culturally tailored outreach, language support, community involvement, education, and empowerment • Leveraging existing resources, grant funding, volunteer support, cost-effective technologies	• A more diverse sample population should be sought • Consideration should be given to potential hemoglobinopathies and medication effects on HbA1c measurements in Bio-Rad samples in the future
Serrano et al*.* [53]	• Difficulty in reaching some subgroups despite extensive outreach, due to cultural and language barriers, limited event attendance, and community trust issues • Logistical challenges with staffing and managing community-based testing, as the setup required a significant number of trained personnel and coordination across multiple community sites	• Collaboration with community organizations to build trust and increase participation • Use of point-of-care testing (POCT) for rapid results and immediate feedback • Providing culturally appropriate education materials • Follow-up with participants through a post- intervention survey to assess lifestyle changes and satisfaction	• Increase scalability by incorporating AI-driven educational tools to reduce the need for on-site educators • Strengthen partnerships with local community organizations for sustainable program delivery • Implement structured long-term follow-up to assess the effectiveness of lifestyle changes
Shemesh et al*.* [54]	-	-	• Further studies with a larger sample size are necessary to validate the performance characteristics of the DCA 2000 in similar settings
Shephard et al*.* [55]	• Managing ongoing diabetes screening and care in rural and remote areas incurs additional costs and geographical challenges	• Utilizing point-of-care offered immediate results to individuals, enhancing patient engagement and retention in disease registers • Local acceptance of point-of-care testing was notable	• Sustaining remote education and training for POCT operators amidst frequent staff turnover is vital as POCT usage expands in rural Australia
Tabaei et al. [56]	• Difficulty in reaching and engaging the target population. • Limited awareness of screening importance. • Cultural and language barriers. • Financial constraints for organizing events, recruiting qualified personnel, coordinating across multiple sites, and securing partnerships with community organizations.	• Standardized protocols ensured consistent and accurate screening, bolstered by collaborative partnerships for improved access to diabetes care. • Engaging voluntary organizations reduced costs and expanded community involvement, optimizing cost-effectiveness.	• Enhanced criteria for defining a positive test. • Targeted outreach to racial and ethnic minorities, and medically underserved populations lacking primary care or insurance could significantly enhance the effectiveness of community-based diabetes screening.
Tantipoj et al. [57]	• Patients with anemia were excluded from the studybased solely on self-reports, without confirmation. • Undiagnosed anemia could potentially affect theprevalence of hyperglycemia.	-	• Patients seeking dental care in the study may have had a higher likelihood of having diabetes mellitus (DM) due to the known association between oral diseases and DM, so findings may not be generalizable to other community settings.
Wensil et al. [58]	• Challenging recruiting and engaging migrant farm workers due to financial constraints and language barriers. • Cost limitations initially impacted screening sample size and follow-up compliance. • Personnel and resource needs for screenings in migrant camps, including Spanish interpreters. • Logistical challenges (i.e. site selection, access tohealthcare facilities, transportation, and the transient nature of the population).	• Securing additional funding for follow-up labwork to improve participation rates. • Using Spanish interpreters and simplified study information to overcome language barriers. • Collaborating with community organizations for outreach and access to healthcare services. • Employing cost-effective point-of-care screening tools for efficiency. • Optimizing personnel deployment for streamlined screening processes.	• Incorporating a health equity framework to address disparities, focusing on improving follow-up strategies for timely access to care and strengthening community engagement and partnerships. • Tailoring interventions to cultural needs and linguistic preferences. • Implementation of long-term monitoring and support programs, with ongoing evaluation of program impact for continuous quality improvement.
Yonel et al. [59]	• Study had a small sample size, and it was logistically challenging to conduct risk assessments without additional available staff.	• The risk-assessment methods utilized appeared to effectively identify individuals at high risk who were previously undiagnosed and unaware of their risk status.	• Community pharmacists and general dental practitioners should conduct risk assessments for type 2 diabetes, ideally with a larger and more ethnically diverse sample size, while also evaluating the cost-effectiveness of theseprograms.

### Collating, Summarizing, and Reporting the Results

**Fig. 2:**
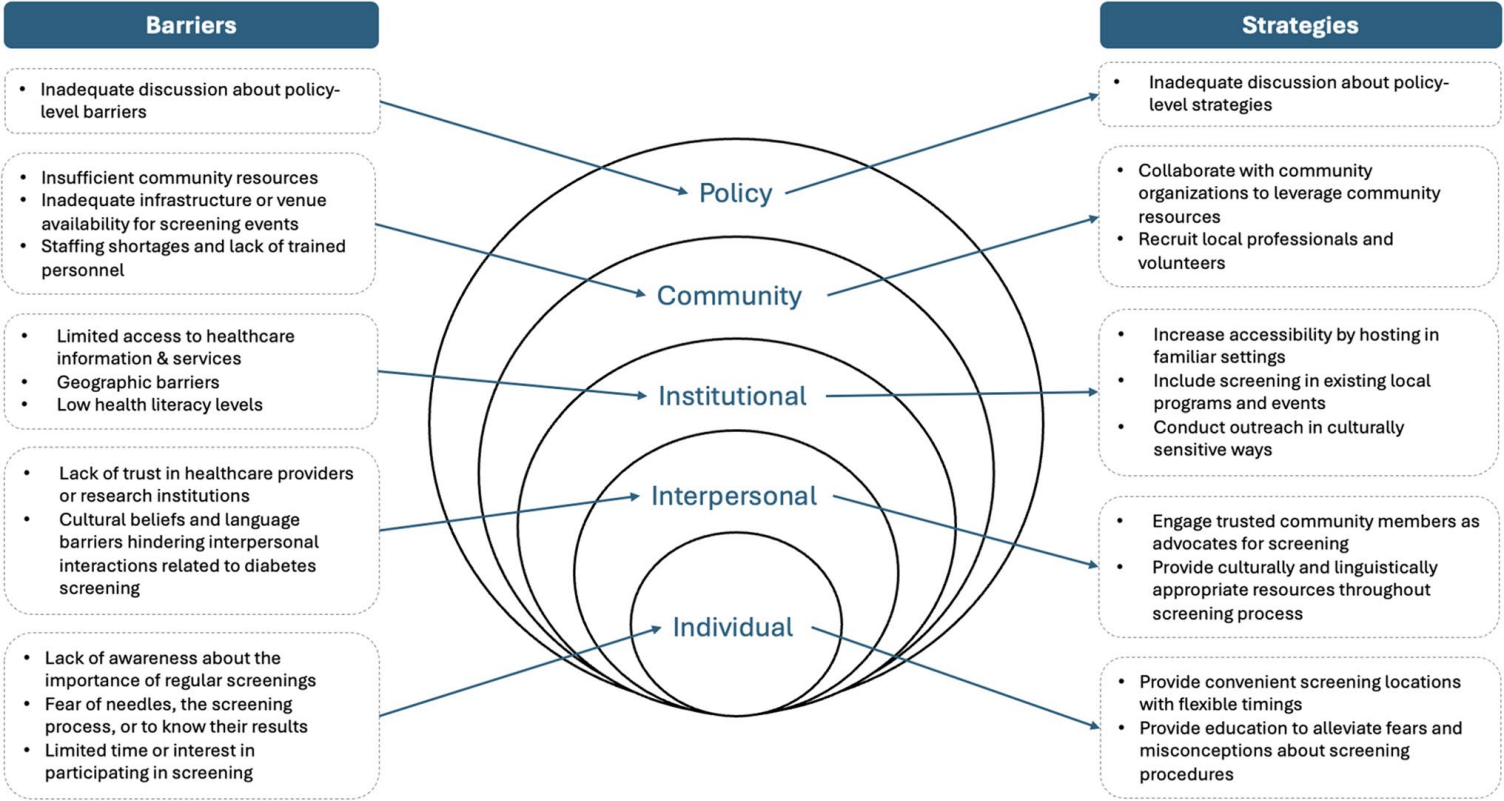
Challenges and strategies reflected in the socioecological model of health

We used the socioecological model of health as the conceptual framework to interpret the results [60]. Descriptive analysis summarized the characteristics of the included studies, while inductive thematic analysis identified key themes related to barriers in T2D screening. These themes were mapped to individual, interpersonal, community, institutional, and policy levels (Fig. 2) [60]. The analysis provides insight into how systemic inequities affect early detection in priority populations and highlights potential areas for future interventions.

### Consultation with Community Partners

A consultation workshop was organized with community partners in Peel Region, Ontario, Canada including WellFort Community Health Services, LAMP Community Health Center, Punjabi Community Health Services, Indus Community Services, Roots Community Services, and Dixie-Bloor Neighbourhood Centre, along with representatives from academic partners as well as health initiatives such as the Novo Nordisk Network for Healthy Populations and provincial policy representatives from Ontario Health. The objectives of the workshop were to share preliminary findings from the scoping review, and interpret them as aligned with the mission and goals of the invited community organizations dedicated to early detection and screening of prediabetes and T2D in community settings; and to gather input for future co-design efforts of such an intervention, a community-based T2D screening intervention, based on recent findings and future directions. During the consultation, participants were presented with preliminary findings from the scoping review, including common barriers and strategies to designing and implementing a community-based intervention that is dedicated to early detection of prediabetes and T2D and connection to the health care system. The participants offered important on the relevance and applicability of the findings from the review to determine how these approaches will meet the needs of the communities they serve as well as future co-design approaches of such an intervention with a particular focus on connecting individuals to the health care system and community support programs. Their feedback helped to contextualize our findings and guide the framing of our recommendations for future community-based T2D screening interventions while considering implementation in real-world settings.

### Ethics Approval

Ethics approval was granted by the University of Toronto’s Ethics Review Office, confirming adherence to ethical guidelines for minimal-risk studies involving literature reviews.

## Results

### Study Characteristics

This review includes diverse study populations, with the majority from the USA (42%, 14/33) [34–36, 39–43, 45–48, 56, 58], followed by Australia (16%, 5/33) [38, 44, 53–55]. Other regions include Europe (16%, 5/33) [31, 37, 50, 51, 59], Canada (9%, 3/33) [27, 49, 52], Africa (6%, 2/33) [30, 32], Asia (3%, 1/33) [57], the Middle East (3%, 1/33) [28], Oceania (3%, 1/33) [29], and South America (3%, 1/33) [33] (Table 1). Targeted populations for screening included pharmacy customers (21%, 7/33) [28, 31, 37, 44, 50, 51, 53, 59], rural residents (16%, 5/33) [33, 38, 40, 47, 54], underserved ethnic communities (42%, 14/33) [29, 34, 38, 42, 45, 46, 48, 49, 52–56, 58], dental patients (16%, 5/33) [35, 36, 41, 57, 59], healthcare center visitors (3%, 1/33) [43], and unhoused individuals (3%, 1/33) [39] (Table 1). Sample sizes varied from 39 to 3,506 participants, with age ranges spanning from 15 to 93 years old (Table 1). Approximately 55% (18/33) of studies included individuals aged 18 and older [29–33, 37, 38, 42, 43, 45, 47, 48, 50, 52–55, 58] (Table 1). All studies included both sexes, except for one [48], and 70% (23/33) had female majority participants [29–31, 33–37, 39, 42–47, 50, 51, 55–57, 59] (Table 1). Several studies explicitly had a health equity focus (i.e., interventions designed to reduce disparities in healthcare access or outcomes), with approximately 61% (20/33) addressing health equity concerns through culturally sensitive approaches, community-based participatory research methods to identify barriers, and tailored interventions recognizing the role social determinants of health in such interventions [29, 31, 33, 34, 38–40, 42, 43, 45–49, 52–56, 58] (Table 1).

### Screening Tools & Personnel

Most studies used biomarkers such as HbA1c, fasting glucose, blood pressure, and BMI, with HbA1c being the most common (88%, 29/33) [27–29, 31, 33–55, 57, 59] (Table 2). The DCA HbA1c Analyzer was the most utilized POC testing tool as presented in 30% (10/33) [36, 38, 46, 49–51, 54, 55, 57, 59], followed by the A1CNow + test in 19% (6/33) [29, 34, 35, 47, 48, 58]. Other devices included the Abbott Affinion TM 2 Analyzer (6%, 2/33) [33, 53], Bio-Rad in2it (3%, 1/33) [52], and Roche Cobas b 101 dual system (6%, 2/33) [28, 31], Accu-Chek, ‘compact’ model (3%, 1/33) [40], CardioChek PA analyzer (3%, 1/33) [30], and Glucomen® Aereo 2 K (3%, 1/33) [37] were mentioned in some studies (Table 2). There was no specified POC device mentioned in 21% (7/33) [27, 32, 39, 42–44, 56] of the studies, and 6% (2/33) [41, 45] mentioned multiple POC devices (Table 2). Personnel conducting screenings varied, with 43% of studies (14/33) using study teams [29, 30, 32, 34, 41, 45–48, 52, 54, 56, 58, 59] and 27% (9/33) using primary healthcare professionals [27, 33, 35, 38, 40, 43, 49, 53, 55]. Other personnel mentioned in the articles included dental providers (3%, 1/33) [36], pharmacists (19%, 6/33) [28, 37, 44, 50, 51, 53], laboratory/research assistants (3%, 1/33) [42], and health professional students (3%, 1/33) [39]. One study, while mentioning collaboration with various dental and medical institutions, did not specify the personnel assigned to the screening [57].

### Screening Settings

Screening activities were conducted across diverse settings, reflecting the community-based approach adopted by many interventions. For example, multiple screening sites (such as parent/teacher night, local fairs, community events, and shopping centers) were mentioned in 36% (12/33) of the studies [30, 33–35, 41, 47, 52, 53, 55–57, 59], followed by community pharmacies (21%, 7/33) [27, 28, 31, 37, 44, 50, 51] (Table 2). Additional sites included dental clinics (3%, 1/33) [36], churches (6%, 2/33) [29, 45], mobile vans (6%, 2/33) [40, 49], barbershops (3%, 1/33) [48], health and social services outreach events (10%, 3/33) [39, 46, 58], public transport hubs (6%, 2/33) [32, 42], and community health centers (3%, 1/33) [43] (Table 2). Other studies lacked a specified screening site (6%, 2/33) [38, 54] (Table 2).

### Screening Process

The screening duration varied from 1 day to 4 years (Table 3). Most studies (70%, 23/33) [27–31, 33, 35–39, 41, 43–45, 47, 50–52, 56–59] combined risk assessment questionnaires with POC testing, while others (27%, 9/33) [32, 34, 40, 42, 46, 48, 49, 54, 55] used only POC testing. Priority populations, including underserved ethnic communities (i.e., immigrant or indigenous communities) [29, 34, 38, 42, 45, 46, 48, 49, 52–56, 58] and unhoused individuals [39], had the highest percentage of individuals meeting criteria for T2D (48.20%), while studies without specific populations had the highest percentage of individuals with prediabetes (61.80%) [27, 28, 30–33, 35–37, 40, 41, 43, 44, 47, 50, 51, 57, 59]. Referrals to primary care providers were common in 89% (29/33) of the studies [27–29, 31–33, 35–51, 53, 55–59], with only 9% (3/33) of studies [38, 47, 53] providing community support program referrals.

#### Challenges and Strategies on the Socioecological Model of Health

Mapping the barriers and strategies of community-based T2D screenings onto the socioecological model of health contextualized how various factors at the individual, interpersonal, institutional, community, and policy levels are interconnected and influence how community-based T2D screening initiatives are developed and implemented (Table 4, Fig. 2).

### Individual Level

Barriers at the individual level included lack of awareness of T2D screening and competing personal priorities (i.e., work or caregiving responsibilities) with other activities in their daily life [28, 32, 33, 46, 48, 58]. There also exists a pervasive reluctance among some individuals to seek out screening services, often due to trust, misconceptions or uncertainties about the screening process and its implications [28, 32, 33, 42, 46, 48, 58]. Fear of needles or receiving unfavorable results further deters individuals from participating in screening initiatives [28, 32, 33, 46, 48, 58].

To mitigate these barriers, some studies involved implementing targeted health education campaigns, through media or word-of-mouth, to raise awareness about the benefits of early detection and the manageable nature of T2D when identified early [32, 38, 42, 45, 46, 48, 50, 52, 56, 58]. Providing culturally sensitive and linguistically appropriate educational materials were also found to enhance the understanding and engagement among diverse populations in early detection of T2D [32, 38, 42, 45, 46, 48, 50, 52, 56, 58]. This also includes offering convenient and accessible screening locations, such as community centers, places of worship, or pharmacies, with extended hours and no appointment or I.D. requirements to encourage participation [27, 31, 34, 42, 45–47].

### Interpersonal Level

Barriers at the interpersonal level included lack of trust, diverse cultural beliefs on T2D screening, and communication barriers that influence screening participation [32, 33, 42, 46, 48, 50, 52, 53, 56]. Mistrust in healthcare, often due to systemic inequalities or negative past experiences, leads to skepticism about screening initiatives and their incentives [32, 33, 42, 46, 48, 50, 52, 53, 56]. Cultural beliefs and language barriers further hinder communication, leading to potential misunderstandings between providers and community members, reducing participation [32, 33, 42, 46, 48, 50, 52, 53, 56].

To address these barriers, interventions focused on building trust and fostering culturally sensitive communication [29, 42, 48, 52, 53]. Engaging trusted community leaders to be advocates, providing tailored education, and offering language interpretation services can improve accessibility [27, 29, 38, 42, 45, 47, 48, 52, 53, 56]. Moreover, open dialogue and safe spaces for addressing concerns can bridge communication gaps and reduce tensions [27, 38, 45, 47, 56]. Creating supportive environments during screening events, characterized by empathetic and nonjudgmental interactions, can help alleviate fears and anxieties, fostering a sense of safety and trust among participants [27, 38, 45, 47, 56].

### Community Level

Community-level challenges included limited resources such as funding, infrastructure, and personnel to coordinate and conduct screenings [29, 33, 36, 38, 46, 51, 53, 55]. Several studies cited limited funding, staffing shortages, venue options, and lack of adequately trained personnel, often obstructed the implementation of screenings [33, 38, 46, 53, 55]. For example, remote screening locations with high staff turnover rates added complexity to community engagement efforts, as these turnover rates could lead to a loss of trusted individuals within the healthcare system, making it harder to build and maintain rapport with community members [38, 42, 54, 55, 59]. Venue availability, especially in underserved areas, and tight schedules at sites such as dental clinics with emergencies or faith-based centers with ceremonies constrained screenings, along with delays in testing kit supplies and maintaining suitable testing conditions [29, 33, 36].

Addressing T2D screening challenges requires strong community engagement and partnerships with local organizations, healthcare providers, and community members or persons with lived experience [29, 42, 48, 52, 53]. These collaborations help recruit and empower local leaders and volunteers, enhance program design, and ensure culturally sensitive and effective screenings [27, 38, 53, 55, 56]. Partnering with communities also leverages established networks and understanding of optimal venues, strategies to reduce costs, and offer support to guide and tailor post-screening efforts based on the needs of the individuals screened [27, 38, 45, 47, 53, 56]. Adopting innovative strategies, such as holding screenings during community events (i.e., school parent/teacher nights) or integrating them with other services (i.e., dental check-ups), can also boost participation and make programs more sustainable and impactful [37, 47, 52, 53, 55, 56].

### Institutional Level

Institutional challenges included disparities in healthcare access (i.e. attachment to primary care), low health literacy, and geographical barriers [29, 31, 33, 36, 38, 46, 51, 55]. Some marginalized communities often encountered lack of availability in routine screenings and preventative care [29, 33, 36, 38, 46, 51, 55]. Such scarcity often resulted in individuals facing significant obstacles when attempting to access T2D screening and preventive care in the community [29, 33, 36, 38, 46, 51, 55]. Gaps in referral pathways between screenings and primary care led to coordination issues and loss of follow-up [30, 32, 58]. Low health literacy and remote locations further hindered individuals’ ability to access and understand the importance of screening services [29, 31, 33, 36, 38, 46, 51, 55].

To address institutional barriers, some interventions brought screening services directly to the community through partnerships with local health organizations and leaders, ensuring culturally appropriate care [29, 30, 32, 42, 46, 48–53, 56]. Resources, such as testing supplies and personnel, were allocated based on community needs, and outreach initiatives such as door-to-door campaigns, collaboration with community organizations for promotions, and workshops attracted participants [29, 30, 32, 42, 46, 48–53, 56]. Non-traditional settings like community pharmacies and mobile vans expanded access, while integrating screenings into routine healthcare activities improved detection rates [31, 33, 35, 43, 59]. By bringing screening services directly to the community, these interventions addressed geographical and infrastructural barriers.

### Policy Level

The studies identified in this review lacked discussion on policy-level barriers and strategies for community-based T2D screening initiatives. Upstream factors such as structural racism, trauma, and systemic oppression [61, 62] that may need to be considered as potential barriers to promote and increase screening efforts among equity-seeking populations were not captured in the included studies in this review. Other key issues such as migration, and unequal distribution of wealth, power, and resources that further drive health disparities and influence a higher burden of T2D were not also mentioned in the studies explored in this review [61, 62].

## Discussion

### Key Findings

This scoping review is the first to understand how community-based T2D screening interventions for early detection of prediabetes and T2D are developed and implemented, offering valuable insights into the key barriers and approaches for implementing such strategies in community settings for diverse populations. The review identified several critical factors, including the need for adequate resources, accessible and familiar settings, and strong community engagement efforts and collaborations for successful community-based T2D screening interventions. The use of POC testing emerged as a practical glucose testing tool for early detection in community settings, providing immediate glucose results that encouraged follow-up care. Additionally, referral pathways and post-screening interventions were essential for ensuring continuity of care, yet challenges in follow-up processes post-screening and evaluation of these interventions remain a concern. However, the effectiveness of these interventions was often contingent on strong referral pathways, culturally tailored educational resources, and addressing structural inequities, such as unstable housing, geographic isolation, and lack of health insurance. As well, in some cases, uninsured individuals could access screening, but faced challenges in obtaining follow-up care. While some programs successfully integrated screening with primary care services and community-based follow-up interventions, many struggled with high attrition rates and a lack of long-term engagement with screened individuals. Importantly, the review also highlighted gaps in the research, particularly around cost-effectiveness analyses and post-screening evaluations, which are necessary to inform future early detection and screening initiatives of prediabetes and T2D.

### The Importance of Funding and Human Resources

Additionally, several studies showed that adequate resources, particularly funding and personnel, are fundamental to the success of community-based T2D screening interventions [29, 36, 39, 42, 45, 46, 53]. Financial investment supports both direct and indirect costs, including POC testing, test kits, staff training, and culturally tailored educational materials and care pathways, all of which are essential for effective implementation [29, 36, 39, 42, 45, 46, 53]. Several studies in this review demonstrated that well-resourced programs were more successful in engaging communities and providing consistent screening [29, 36, 39, 42, 45, 46, 53]. Personnel, including healthcare workers and community outreach staff, played a critical role in delivering services, educating participants, and ensuring follow-up pathways [29, 36, 39, 42, 45, 46, 53]. Their involvement was instrumental in maintaining trust and continuity in the screening process, particularly within underserved populations [29, 36, 39, 42, 45, 46, 53].

### Point-of-Care Screening Tools

Furthermore, the value of community-based T2D screening programs that used POC testing provided a significant avenue for future early detection interventions [27, 34, 42, 45–47, 53]. POC testing tools, such as HbA1c analyzers, were commonly used in the studies, offering immediate glucose results [42, 44, 53]. The immediate availability of these tests encouraged greater adherence to follow-up care [42, 44]. Moreover, the portable nature of POC testing devices allowed for its use in diverse settings where screenings could meet “individuals where they were,” contributing to increased participation and ability to screen more diverse populations [27, 34, 42, 45–47, 53]. Future interventions could leverage these tools to provide efficient and effective screening, particularly in underserved communities where traditional healthcare access is limited.

### Settings for Community-Based T2D Screening Interventions

The choice of setting and timing for community-based T2D screenings significantly affected participation rates [27, 31, 34, 42, 45–47, 53]. Screenings held in accessible, community-centered locations, such as faith-based and community centers, and pharmacies, where individuals already felt comfortable were more likely to engage participants [27, 31, 34, 42, 45–47, 53]. Offering screenings in settings that aligned with daily routines reduced barriers to participation, as individuals could integrate screenings into their existing schedules [27, 31, 34, 42, 45–47, 53]. Similarly, providing screenings at convenient times, such as evenings or weekends, increased accessibility for working individuals and those with time constraints [27, 31, 34, 42, 45–47, 53].

### Building Community Partnerships and Trust

Moreover, effective and meaningful community engagement efforts with local organizations, community leaders, and institutions were critical to the success of screening interventions [27, 29, 30, 32, 42, 46, 48–53, 56]. Engaging trusted community leaders as advocates, incorporating culturally tailored education, and offering language interpretation services further improved accessibility, particularly for linguistically diverse communities [27, 29, 38, 42, 45, 47, 48, 52, 53, 56]. Collaborations with trusted community figures, such as community leaders, religious leaders, or local health providers, helped tailor programs to meet specific community needs [27, 29, 30, 32, 42, 46, 48–53, 56]. These partnerships also built credibility and fostered trust, especially within priority populations that may have harbored skepticism toward healthcare initiatives [27, 29, 30, 32, 42, 46, 48–53, 56]. By leveraging existing community networks, these partnerships not only extended the reach of interventions but also enhanced their acceptance and integration into the community [27, 29, 30, 32, 42, 46, 48–53, 56]. To further strengthen these efforts, engaging community members in program design and evaluation is critical to ensuring interventions address unmet needs. Studies that utilized community-based participatory approaches, community advisory boards, peer educators, and local leadership demonstrated promising strategies for meaningful participation [27, 29, 38, 42, 45–47, 49, 53, 55]. Establishing these collaborations and building trust may also support efforts to identify why certain populations are not represented in community-based T2D screening interventions. For instance, our reviewed showed that several studies observed a predominance of female participants in T2D screening programs [29–31, 33–37, 39, 42–47, 49–53, 55–57, 59], and while this may be attributed to gender-specific health-seeking behaviours influencing participation rates for certain genders, this also highlights the need for more inclusive, gender-sensitive approaches to increase engagement across all demographics.

### Strengthening Referral Pathways and Follow-Up Care

A major finding from this review was that well-structured referral pathways connecting individuals to primary care and community support are essential for ensuring long-term engagement and improved health outcomes in community-based T2D screening interventions [29, 34, 36, 38, 39, 42, 46–48]. However, many programs lacked formalized systems to ensure sustained follow-up and care coordination, limiting their long-term effectiveness [28, 29, 32, 35, 58]. Tailoring referral pathways to meet individual needs was found to be vital, particularly for individuals disconnected from the health care system [29, 34, 36, 39, 42, 46, 48]. Follow-up communications, such as phone calls or text reminders, facilitated appointment scheduling and improved adherence [29, 34, 36, 39, 42, 46, 48]. Additionally, linking patients to community support programs offering health behaviour change resources was considered integral for future screening interventions [38, 47, 53]. Thus, this review emphasized that T2D screening interventions with a referral component to connect individuals to primary care, community support services and/or programming is needed to support early diagnoses and care for T2D [38, 47, 53].

Nonetheless, sustaining long-term health care engagement remains a significant challenge. While some studies reported tracking follow-up 12 months [35, 36, 55], most interventions either lacked follow-up data entirely [27, 29–31, 33, 34, 37–41, 43, 47–49, 52, 54, 56, 57, 59] or only reported short-term engagement of less than six months [28, 32, 42, 44–46, 50, 51, 53, 58]. This highlights a gap in understanding the long-term impact of T2D community-based screening interventions. Even among studies that documented initial referrals, follow-up rates varied widely, with some interventions successfully linking over half of screened participants to healthcare providers [28, 32, 35, 36, 42, 44, 46, 53, 58]. Yet, in several of these cases, less than 40% of referred individuals sought care, and many were lost to follow-up entirely [28, 29, 32, 35, 53, 58]. Structural inequities such as unstable housing, geographic isolation, and lack of health insurance, and geographic isolation, along with attitudinal factors such as low perceived urgency contributed to preventing individuals from seeking post-screening care [28, 33, 37–39, 42, 45–47, 53, 58]. For those who did access healthcare services, few studies reported sustained metabolic improvements such as glycemic control, weight management, or cardiovascular risk reduction [29, 37, 38, 46, 55, 58]. While some interventions documented modest reductions in HbA1c and BMI, most lacked long-term tracking beyond initial follow-up visits [28, 35, 36, 55]. Without structured monitoring systems of referral and follow-up pathways, it remains unclear whether these early detection efforts translated into lasting health benefits [32, 42, 56].

### Sustainability and Integration with Health Systems

The long-term sustainability of community-based T2D screening programs presents another critical challenge, particularly regarding funding, integration with healthcare systems, and workforce capacity [27–39, 42, 43, 45, 46, 48–57, 59]. Similar to program development, sustainability is influenced by adequate funding, strong community and health system partnerships, and potential integration of screening efforts into healthcare systems through referral pathways [27–39, 42, 43, 45, 46, 48–57, 59]. Government and institutional funding are also essential to maintain staffing, resources, and follow-up services, particularly for interventions targeting priority populations [27, 28, 30, 32, 33, 36, 37, 39, 45, 49–56, 59]. Furthermore, strategic partnerships with primary care networks, pharmacies, and community organizations may improve accessibility to screenings and facilitate effective referral pathways, ensuring that individuals receive timely follow-up care [28, 30, 32, 33, 36, 37, 39, 45, 50–56, 59]. As well, embedding screenings into routine healthcare visits and public health initiatives, such as national T2D prevention programs, can increase program sustainability and reduce reliance on short-term funding sources [27, 30, 32–36, 42, 45, 47–49, 55–57, 59]. Future research should aim to determine more standardized tracking frameworks and explore scalable models for sustaining community-based T2D screening efforts.

### Innovative Strategies for Improving Continuity of Care

Enhancing continuity of care requires proactive interventions beyond passive referrals. Studies have highlighted the role of community health workers (CHWs) and digital health solutions as promising strategies to bridge gaps between initial screening and long-term healthcare engagement [29, 42, 46, 55, 56]. CHWs play a crucial role in providing culturally tailored education, facilitating follow-up appointments, and guiding patients through the healthcare system, particularly for priority populations [29, 38, 42, 46]. Community-led initiatives, such as community advisory groups and mobile healthcare services, have also shown promise in participant retention and engagement with ongoing care [38, 53, 55]. Additionally, digital health tools, including web-based data tracking, automated appointment reminders, and pharmacy-based monitoring systems, have demonstrated success in improving follow-up adherence in certain interventions [29, 32, 35, 44]. One study proposed incorporating AI-driven educational support to reduce the burden on healthcare staff and enhance scalability in community settings [53]. However, few studies have assessed the long-term effectiveness of either CHW-led or technology-driven approaches, underscoring the need for future research to evaluate their sustainability and scalability [32, 42, 44, 53]. Integrating CHWs with structured patient navigation services, electronic health records, AI-driven educational support and digital follow-up methods may provide a more comprehensive model to enhance continuity of care beyond the initial screening [29, 32, 46, 53, 55, 56].

### Knowledge Gaps and Areas for Future Research

Nonetheless, several knowledge gaps emerged from this scoping review, each presenting opportunities for further community-based T2D screening research and screening intervention development. Firstly, there was a notable gap in the assessment of cost-effectiveness analyses for T2D screening interventions, with few studies exploring their economic implications; future research should focus on evaluating the financial viability and sustainability of these programs to guide resource allocation and decision-making [61, 62]. Secondly, post-screening community programming, such as behavioural modification and ongoing support, was often lacking, which are vital for helping at-risk individuals make sustainable changes and improve their health outcomes [63, 64]. Thirdly, the absence of data on participants'previous screening history and on long-term health engagement limited the understanding of the effectiveness and reach of such screening programs; addressing this gap could enhance the targeting and impact of future interventions.

### Addressing Policy-Level and Structural Barriers

A significant knowledge gap existed in addressing policy-level barriers, such as structural racism, trauma, and systemic oppression, which affect the effectiveness and implementation of T2D screening interventions [65, 66]. These upstream factors perpetuate health disparities emphasizing the need for policies that focus on dismantling these upstream or macro-level barriers and promote equitable access to early detection and screening programs for T2D [61, 65, 66]. To address these gaps, it is essential to advocate for policy changes that reduce structural inequities. This may include creating targeted public health strategies that address social determinants of health, such as poverty, lack of access to healthcare, and inadequate housing [61, 65, 66]. Collaborating with community-based organizations to co-design inclusive policies can ensure that screening initiatives address the lived experiences of marginalized populations [65, 66]. Embedding health equity principles within public health frameworks can further support accessible screening programs [65, 66]. Successful macro-level policy initiatives, such as federally funded frameworks and community health strategies, have demonstrated potential in expanding screening access [67, 68]. For example, the Framework for Diabetes in Canada was recently developed in accordance with *the National Framework for Diabetes Act* to provide policy directions to address T2D in Canada through various components [67, 68]. One of the components include *Prevention*, which emphasizes the underlying social and environmental conditions and the upstream risk factors that influence T2D risk and are rooted in sectors beyond health [67, 68]. As part of this component, it is recommended that T2D prevention efforts should “*promote, support and widely utilize validated preventative measures (e.g., screening tools to determine T2D risk)”* [67, 68]*.* Therefore, community-based T2D screening programs can operationalize this component of the Framework to detect prediabetes or T2D early and determine risk, prompt health behaviour changes, and support individuals who are at risk of developing or living with T2D [67, 68]. Aligning screening efforts with broader health policy initiatives can enhance long-term sustainability by securing funding and integrating programs within healthcare systems [65–68]. Building partnerships between healthcare providers, community organizations, and government fosters success, as it ensures policies are contextually relevant, while also enhancing community buy-in and sustained engagement [65–68].

### An Integrated Approach to Community-Based T2D Screening

This scoping review revealed a diverse range of community-based T2D screening strategies, including those tailored for priority populations in underserved ethnic communities and unhoused individuals [29, 34, 38, 39, 42, 45, 46, 48, 49, 52–56, 58]. Such targeted approaches identified a higher prevalence of undiagnosed T2D, highlighting the need for more tailored strategies that identify high-risk groups earlier [29, 34, 38, 39, 42, 45, 46, 48, 49, 52–56, 58]. In contrast, other strategies that were not targeted to specific populations identified higher rates of prediabetes in the community, suggesting a broader need for community-wide preventive measures [27, 28, 30–33, 35–37, 40, 41, 43, 44, 47, 50, 51, 57, 59]. Therefore, this review recommends an integrated approach to community-based T2D screening. Targeted strategies should focus on priority populations, as these efforts can identify undiagnosed T2D earlier and reduce health disparities. Meanwhile, community-wide non-specific population initiatives prove to be effective for detecting cases of prediabetes and supporting preventive care. Future research will need to explore how these programs can be scaled up to determine more sustainable, feasible, effective, and equitable approaches to integrating community-based T2D screening with existing initiatives offered by health systems. For example, embedding T2D screening within primary care networks, pharmacies, and community health centers may enhance accessibility and sustainability of such programs [27, 30, 32–36, 42, 45, 47–49, 55–57, 59]. As more undiagnosed individuals are identified, ensuring timely integration into the healthcare system is critical for initiating early treatment, preventing complications, and ultimately reducing the long-term burden on healthcare resources. While further evidence is needed to develop, test, and evaluate how community-based T2D screening strategies can be linked with existing health care and community health initiatives, other factors such as funding, capacity and resources, as well as political “buy in” need to be considered for potential scaling in other jurisdictions and populations.

### Study Limitations

While this is the first scoping review, to our knowledge, that provides evidence on the design and implementation of community-based T2D screening interventions, it is important to discuss a few limitations. First, this review only included English-language studies, which may have excluded potential non-English studies. Second, despite a comprehensive search strategy across multiple databases and grey literature sources, some relevant studies may have been missed due to indexing differences and inconsistent reporting practices. Third, variations in study designs, screening methodologies, and reporting formats presented challenges to synthesizing findings for all studies to draw direct comparisons across interventions. Without standardized reporting, extracting consistent data on follow-up and implementation presented challenges to synthesize the evidence. Additionally, this review did not include a formal study appraisal or risk of bias evaluation. As a result, study quality and potential biases could not be systematically evaluated.

## Conclusion

Our scoping review supports existing research emphasizing the need for targeted early detection of prediabetes and T2D to promote preventive measures in primary care and community settings, particularly for priority populations [61, 62, 69]. It reinforces the consensus on the importance of tailored, equitable, and sustainable community-based T2D screening approaches and expands the understanding of community-based interventions beyond traditional clinical settings [61–64, 69]. While previous studies have examined community versus facility-based screening [70, 71], our first-of-its kind review explored a broader range of community-based interventions targeting T2D detection and identified key knowledge gaps, particularly in cost-effectiveness analyses and post-screening programming, to guide future research. By emphasizing the development and implementation of such interventions, our review adds valuable insights into early detection community-based and for T2D led strategies that go beyond traditional screening methods [72, 73]. A comprehensive approach to community-based screening requires adequate resources, strong partnerships with community organizations, equitable access to screening sites, and well-established referral pathways to facilitate early detection and prevention of T2D-related complications. POC glucose tests provide immediate and reliable results, improving follow-up adherence and engagement in care. However, this review highlights the need for a systematic evaluation of POC tests to assess their effectiveness, scalability, and integration within healthcare systems. Furthermore, policy-level barriers, such as systemic racism, must be addressed to ensure screening interventions are equitable, feasible, and sustainable. These findings are vital for shaping future community-based T2D screening strategies, fostering early detection, prevention, and long-term health outcomes.

## Key References

- Duong D, Vogel L. National survey highlights worsening primary care access. CMAJ. 2023 Apr 24;195(16):E592–3.
  - This article highlights the barriers that are posed by a declining access to primary care services. The findings elaborate on how the reduced access impacts the early detection and management of chronic diseases, including T2D. This provides context for the urgency and importance of community-based alternatives to traditional healthcare settings, particularly for underserved populations facing systemic inequities in healthcare access.
- Kerkhoff AD, Rojas S, Black D, Ribeiro S, Rojas S, Valencia R, et al. Integrating Rapid Diabetes Screening Into a Latinx Focused Community-Based Low-Barrier COVID-19 Testing Program. JAMA Netw Open. 2022 May 2;5(5):e2214163.
  - This article showcases the feasibility and effectiveness of integrating a T2D screening program into a low-barrier COVID-19 testing initiative successfully reaching Latinx communities, identified as a historically underserved population. Its emphasis on cultural sensitivity, accessibility, and dual-purpose healthcare models can serve as an outline for designing multifaceted community health programs that prioritize equity and efficiency.
- Kim MM, Kreider KE, Padilla BI, Lambes K. Implementation of a Prediabetes Risk Test for an Underserved Population in a Federally Qualified Health Center. Clin Diabetes. 2022;41(1):102–9.
  - This article demonstrates the practical application of prediabetes risk testing in a Federally Qualified Health Center serving underserved populations. The program’s success in identifying at-risk individuals in a low-resource setting underscores the potential of simple, scalable screening tools in bridging healthcare disparities. It also highlights the critical role of community-based healthcare centers in providing preventive care and fostering long-term health improvements.
- Krass I, Twigg MJ, Mitchell B, Wilson F, Mohebbi M, Trinder P, et al. Participant and GP perspectives and experiences of screening for undiagnosed type 2 diabetes in community pharmacy during the Pharmacy Diabetes Screening Trial. BMC Health Serv Res. 2023 Dec 1;23:1337.
  - This article elaborates on the unique and underutilized role of community pharmacies as accessible and trusted sites for T2D screening. It provides valuable insights into participant and provider experiences, demonstrating the potential to scale community-based interventions in community pharmacy settings as a practical and sustainable solution to reach diverse populations. The study reinforces the importance of integrating screening into everyday community interactions to reduce undiagnosed T2D rates.
- Fazli GS, Booth GL. Call for Action on the Upstream Determinants of Diabetes in Canada. Canadian Journal of Diabetes. 2023 Oct 1;47(7):618–24.
  - This article focuses on systemic social determinants of health, such as socioeconomic inequities and systemic racism, that perpetuate T2D disparities. It advocates for upstream policy changes, such as improving social infrastructure and addressing systemic barriers, to reduce T2D incidence. This aligns with the review’s emphasis on addressing root causes of health inequities, making it essential for learning how policies need to be shaped to promote equity in T2D prevention and care.

## Supplementary Information

Below is the link to the electronic supplementary material.

Supplementary file1 (JPEG 1172 KB)

Supplementary file2 (PDF 63 KB)

## Data Availability

No datasets were generated or analysed during the current study.
