# Supplementary figures and images for: A Scoping Review on Community-based Diabetes Screening Interventions: Paving the Pathway to Early Care and Prevention of Diabetes
Source: Curr Diab Rep. 2025 Oct 4;25(1):51. doi: 10.1007/s11892-025-01605-2 (PMC12496278; doi:10.1007/s11892-025-01605-2)

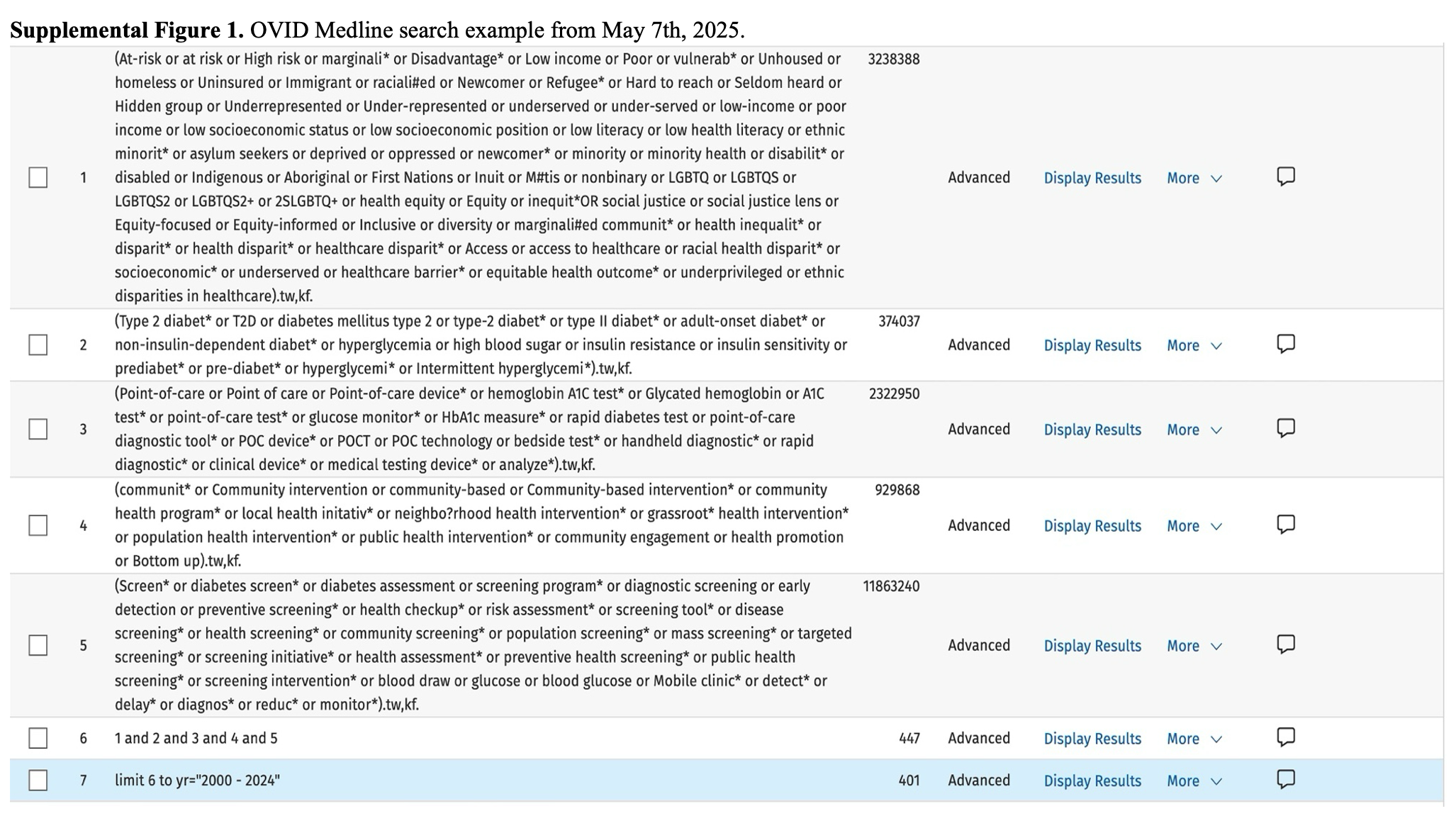

Supplement: Supplementary file 1 — Supplementary file1 (JPEG 1172 KB) [file 11892_2025_1605_MOESM1_ESM.jpeg]
