# Supplementary material for: A Scoping Review on Community-based Diabetes Screening Interventions: Paving the Pathway to Early Care and Prevention of Diabetes
Source: Curr Diab Rep. 2025 Oct 4;25(1):51. doi: 10.1007/s11892-025-01605-2 (PMC12496278; doi:10.1007/s11892-025-01605-2)
Supplement: Supplementary file 2 — Supplementary file2 (PDF 63 KB) [file 11892_2025_1605_MOESM2_ESM.pdf]

Supplemental Table 1. Search Strategy

**Subject Headings**	**Keywords used in database search**	**Reduced keywords used in Grey Literature search**
Population: Equity-denied communities	(At-risk OR at risk OR High risk OR marginali* OR Disadvantage* OR Low income OR Poor OR vulnerab* OR Unhoused OR homeless OR Uninsured OR Immigrant OR raciali#ed OR Newcomer OR Refugee* OR Hard to reach OR Seldom heard OR Hidden group OR Underrepresented OR Under-represented OR underserved OR under-served OR low-income OR poor income OR low socioeconomic status OR low socioeconomic position OR low literacy OR low health literacy OR ethnic minorit* OR asylum seekers OR deprived OR oppressed OR newcomer* OR minority OR minority health OR disabilit* OR disabled OR Indigenous OR Aboriginal OR First Nations OR Inuit OR M#tis OR nonbinary OR LGBTQ OR LGBTQS OR LGBTQS2 OR LGBTQS2+ OR 2SLGBTQ+ OR health equity OR Equity OR inequit* OR social justice OR social justice lens OR Equity-focused OR Equity-informed OR Inclusive OR diversity OR marginali#ed communit* OR health inequalit* OR disparit* OR health disparit* OR healthcare disparit* OR Access OR access to healthcare OR racial health disparit* OR socioeconomic* OR underserved OR healthcare barrier* OR equitable health outcome* OR underprivileged OR ethnic disparities in healthcare).tw,kf.	-
Type 2 diabetes or prediabetes	(Type 2 diabet* OR T2D OR diabetes mellitus type 2 OR type-2 diabet* OR type II diabet* OR adult-onset diabet* OR non-insulin-dependent diabet* OR hyperglycemia OR high blood sugar OR insulin resistance OR insulin sensitivity OR prediabet* OR pre-diabet* OR hyperglycemi* OR Intermittent hyperglycemi*).tw,kf.	Undiagnosed AND diabet*
Point-of-care devices	(Point-of-care OR Point of care OR Point-of-care device* OR hemoglobin A1C test* OR Glycated hemoglobin OR A1C test* OR point-of-care test* OR glucose monitor* OR HbA1c measure* OR rapid diabetes test OR point-of-care diagnostic tool* OR POC device* OR POCT OR POC technology OR bedside test* OR handheld diagnostic* OR rapid diagnostic* OR clinical device* OR medical testing device* OR analyze*).tw,kf.	Point-of-care
Community-based interventions	(communit* OR Community intervention OR community-based OR Community-based intervention* OR community health program* OR local health initativ* OR neighbo?rhood health intervention* OR grassroot* health intervention* OR population health intervention* OR public health intervention* OR community engagement OR health promotion OR Bottom up).tw,kf.	Communit*
Screening programs	((Screen* OR diabetes screen* OR diabetes assessment OR screening program* OR diagnostic screening OR early detection OR preventive screening* OR health checkup* OR risk assessment* OR screening tool* OR disease screening* OR health screening* OR community screening* OR population screening* OR mass screening* OR targeted screening* OR screening initiative* OR health assessment* OR preventive health screening* OR public health screening* OR screening intervention* OR blood draw OR glucose OR blood glucose OR Mobile clinic* OR detect* OR delay* OR diagnos* OR reduc* OR monitor*).tw,kf.	Screen* OR detect*
